# Young apple polyphenol extract prevents diabetic cognitive dysfunction by promoting TET2-mediated active DNA demethylation in the brain of diabetic mice

**DOI:** 10.3389/fnut.2025.1580775

**Published:** 2025-05-02

**Authors:** Jiacheng Fang, Xiaochen Zhang, Zhongshi Qi, Liying Zhou, Yuntiao Chen, Qingxin Li, Run Liu, Hongzhuan Yu

**Affiliations:** ^1^Weifang Hospital of Traditional Medicine, Affiliated Traditional Chinese Medicine of Shandong Second Medical University, Weifang, Shandong, China; ^2^School of Public Health, Qingdao University, Qingdao, Shandong, China; ^3^Department of International Management, Business School, The University of Adelaide, Adelaide, SA, Australia; ^4^Center for Disease Control and Prevention of Tangshan City, Tangshan, Hebei, China

**Keywords:** diabetic cognitive dysfunction, active DNA demethylation, ten-eleven translocation protein 2, young apple polyphenol extract, mitochondrion

## Abstract

**Introduction:**

Diabetic cognitive dysfunction (DCD) refers to the impairment of cognitive function resulting from diabetes. The increasing prevalence of diabetes and the aging population have rendered DCD a significant threat to brain health. Young apple polyphenol extract (YAPE) has demonstrated potential in preventing DCD, although its underlying mechanism remains incompletely understood. Therefore, this study aims to investigate the preventive efficacy and underlying mechanisms of YAPE on DCD.

**Methods:**

Streptozotocin-induced diabetic mice were randomly divided into four groups (*n* = 15): the diabetes model group (DM), the metformin group (MET), the low-dose young apple polyphenol group (LYAP), and the high-dose young apple polyphenol group (HYAP). Meanwhile, 15 additional mice were assigned as the control group (CON).

**Results:**

Following a 14-weeks intervention, disrupted cognitive function and neuronal apoptosis were observed in DM group, both of which were effectively restored by YAPE supplementation through improving ten-eleven translocation protein 2 (TET2)-mediated active DNA demethylation. Moreover, YAPE supplementation enhanced TET2 protein stability by activating phosphorylated AMP-activated protein kinase (AMPK) and improved TET enzyme activity by upregulating α-ketoglutarate/(succinic acid + fumaric acid) ratio, subsequently enhancing TET2 function.

**Discussion:**

Consequently, YAPE effectively delays progression from diabetes to DCD by facilitating TET2-mediated active DNA demethylation.

## Graphical abstract

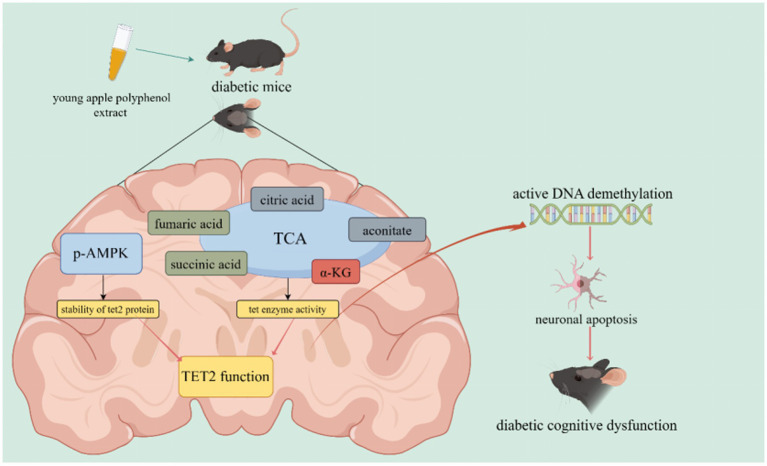

## 1 Introduction

Diabetic cognitive dysfunction (DCD) refers to the impairment of cognitive function induced by diabetes, clinically manifested as reduced capabilities in language, visual memory, information processing speed, and linguistic ability (1). At the end of 2021, approximately 529 million individuals worldwide were living with diabetes, and its prevalence remains persistently high (2). Epidemiological studies have revealed that the prevalence of DCD among individuals with diabetes is approximately 20–30%, with a conversion rate to dementia reaching 17.3% (1, 3). Another cohort study demonstrated that the risk of developing dementia among individuals with diabetes is approximately 2.8 times higher compared to their non-diabetic counterparts of the same age and gender (4). DCD has become a significant public health concern. Therefore, elucidating the underlying mechanisms of DCD and exploring effective prevention and intervention strategies is important to public health.

Our previous study indicated that the inhibition of ten-eleven translocation 2 (TET2)-mediated active DNA demethylation represents a potential mechanism underlying the pathogenesis of DCD (5). The regulation of TET2 function is governed by its protein stability and enzymatic activity. It is well known that α-ketoglutarate (α-KG), an intermediate of the tricarboxylic acid (TCA) cycle, serves as a cofactor for TET enzymes (6). Conversely, succinic acid and fumaric acid, two other TCA products, act as competitive inhibitors of TET enzymatic activity by competing with α-KG (7, 8). Furthermore, AMP-activated protein kinase (AMPK) enhances the stability of TET2 by phosphorylating it at the serine 99 residue within the TET2 protein (9). The accumulating body of evidence suggests that the upregulation of TET2 function can be achieved by activating AMPK phosphorylation and enhancing mitochondrial function in the brains of diabetic mice, thereby exerting inhibitory effects on diabetes-induced neuronal apoptosis (10–12). Therefore, we propose that nutrients capable of activating AMPK and maintaining TCA homeostasis possess potential efficacy in preventing the onset of DCD by stimulating TET2-mediated active DNA demethylation.

Epidemiological studies have supported strong associations between the dietary intake of apples or apple-related products and a decreased risk of diabetes, cardiovascular disease, and cognitive dysfunction (13, 14). Thinned young apple extract has garnered significant attention due to its anti-inflammatory and antioxidant properties (15, 16). Approximately 2 million tons of young apple by-products generated during fruit thinning are annually discarded in Chinese fields, leading to significant resource wastage and environmental pollution (17). The polyphenol content of young apples is approximately 10 times higher than that of ripe apples. Young apple polyphenol extract (YAPE) is a mixture of polyphenols obtained from the entire thinned young apples, with chlorogenic acid, phloridzin, and hyperoside as its main constituents (18). Meanwhile, it has been confirmed that apple polyphenol extract enhances cognitive function by inhibiting neuroinflammation and prevents neuronal apoptosis by upregulating 5hmC in the brains of mice (19, 20). However, no studies have investigated the preventive effects of YAPE on DCD and its underlying molecular mechanisms. Therefore, in the present study, we conducted cognitive behavior tests on streptozotocin-induced diabetic mice to evaluate the impact of YAPE supplementation on cognitive function, with the aim of validating its preventive efficacy against diabetic cognitive dysfunction (DCD). In addition, we investigated DNA epigenetic modification levels and DNA epigenetic regulatory factors in the brain to elucidate the molecular mechanism underlying YAPE’s preventive effect on DCD.

## 2 Materials and methods

### 2.1 Extract, separation, and determination of YAPE

The extraction, purification, and determination protocols for YAPE were performed according to our previously established methods (16, 21). In brief, the experimental procedure consisted of the following key steps: Young apples were first finely ground into particles ranging from 3 to 4 mm in size. These particles were then immersed in a 60% ethanol solution and subjected to continuous extraction for 3 h at 65°C using an oscillatory device. Subsequently, the filtration process was conducted using quantitative filter paper and a Buchner funnel. Ethanol was removed by rotary distillation to concentrate the extract. The concentrated solution was centrifuged at 3,500 g for 20 min, and the supernatant was freeze-dried to obtain YAPE powder. The total polyphenol content was determined using the Folin–Ciocalteu method and expressed in gallic acid equivalents. Moreover, for precise quantification of monomeric phenols, a high-performance liquid chromatography system (Thermo Ultimate 3000) was used, with quantification based on peak area calculations.

### 2.2 Animals and experimental design

The animal study protocol was approved by the Ethics Committee of the Medical College of Qingdao University (No. QDU-AEC-2022468). In brief, 80 8-week-old male C57BL/6J mice were obtained from Beijing Vital River Laboratory Animal Technology Co., Ltd. and housed at the animal experimental center of Qingdao University. The environmental conditions were carefully controlled, maintaining temperatures between 23 and 25°C, humidity levels between 55 and 65%, and a 12-h light/12-h dark cycle. Throughout the experiment, the mice had unrestricted access to food and water, and their body weight was recorded on a weekly basis.

After 1 week of adaptive feeding, 65 mice were randomly selected to receive daily peritoneal injections of streptozotocin (STZ) at a dosage of 55 mg/kg for 5 consecutive days. Meanwhile, the remaining 15 mice were assigned to the control group (CON) and received peritoneal injections of an equivalent volume of saline solution. After STZ injection, the mice underwent fasting blood glucose (FBG) testing, and those with FBG levels > 11.1 mmol/L were diagnosed as diabetic and subsequently included in a 12-week intervention trial. In the present study, 60 diabetic mice were randomly divided into four groups (*n* = 15): the diabetes model (DM) group, the metformin (MET) group, the low-dose young apple polyphenol (LYAP) group, and the high-dose young apple polyphenol (HYAP) group. The DM group received normal saline, while the MET group was administered metformin solution at a dosage of 150 mg/kg. The LYAP group was administered YAPE at a dosage of 150 mg/kg, while the HYAP group was administered YAPE at a dosage of 300 mg/kg. During the intervention, the mice in the CON group were provided with a standard diet (10% kcal from fat content, D12450J), while the mice in the other four groups were provided with a high-fat diet (60% kcal from fat content, D12492). After completing the intervention, the mice underwent an overnight fast before being killed. Blood samples were collected from the posterior orbital sinus for serum biochemical measurements. Brain tissue for histological analysis was obtained following cardiac perfusion with saline and subsequently fixed in 4% paraformaldehyde. Brain tissue samples intended for biochemical detection were collected, rinsed with saline, and promptly frozen in liquid nitrogen.

### 2.3 Behavioral test

The cognitive function of the mice was assessed using the Morris water maze, Y-maze, and novel object recognition (NOR) tests during the 12th week of the intervention. The detailed experimental protocol has been previously described in our published studies (5, 22). The mice were given a 1-day rest after completing both the Y-maze and NOR tests.

### 2.4 Intraperitoneal glucose tolerance test

The intraperitoneal glucose tolerance test (IPGTT) was conducted during the 14th week of the intervention. The mice underwent an 8-h fasting period, after which a glucose solution (2 mg/kg) was administered via intraperitoneal injection. Blood samples were collected from the caudal vein at 0, 30, 60, 90, and 120 min post-injection for glucose concentration analysis using a blood glucose meter (Roche Active).

### 2.5 Serum biochemical parameters, dot blot, and Western blot

The levels of insulin in the mouse serum were measured using a commercial ELISA kit following the manufacturer’s protocol (Jingkang, Shanghai, China). The homeostasis model assessment of insulin resistance (HOMA-IR) was calculated using the following formula: (fasting insulin concentration × fasting glucose concentration × 0.05551)/22.5. The concentrations of total cholesterol (TC), triglyceride (TG), low-density lipoprotein (LDL), and high-density lipoprotein (HDL) in the mouse serum were measured using an automatic biochemical analyzer (Sysmex XN-10). The levels of S-adenosylmethionine (SAM) and S-adenosylhomocysteine (SAH) in the cerebral cortex were examined using an ELISA kit following the manufacturer’s protocol (Huijia Biology Company, Guangzhou, China). Genomic DNA from the cerebral cortex was isolated using the TIANamp Genomic DNA Kit according to the manufacturer’s instructions (Tiangen, China), and the concentration of DNA was determined using a NanoDrop spectrophotometer (Thermo Scientific). The details of the dot blot and Western blot protocols have been described in our previous studies (5, 11, 12).

### 2.6 Detection of TCA metabolites in the cerebral cortex of mice

We used the Agilent 1290 Infinity LC system coupled with the Agilent 6530 Precision Mass Quadrupole Time-of-Flight (Q-TOF) system (Agilent, United States) to quantify tricarboxylic acid (TCA) cycle intermediates. During the pretreatment phase, 30 mg of cerebral cortex tissue was added to 100 μL of an extraction buffer (acetonitrile: methanol: water = 2:2:1, precooled at −20°C) and adequately ground with a homogenizer (24 × 2, 70 Hz, 60 s, twice). The homogenized samples were incubated at −80°C overnight to allow for complete protein precipitation. Following sedimentation, 100 μL of acetonitrile containing 0.2% acetic acid was added, and the solution was thoroughly vortexed. This was followed by centrifugation at 4°C for 10 min at 13,000 rpm. A 150 μL aliquot of the supernatant was then transferred to a sample bottle for analysis. Chromatographic separations were performed using a ZORBAX SB-C18 column (2.1 mm × 100 mm, 5 μm, Agilent, United States) and maintained at 35°C. The mobile phase consisted of water with 0.1% formic acid (solvent A) and methanol with 0.1% formic acid (solvent B). The gradient elution protocol proceeded as follows: 20% B at 0–2 min, 25% B at 4 min, 30% B at 6 min, 90% B at 10–12 min, and 20% B at 12.1–15 min. The flow rate was 0.7 mL/min, and the injection volume was 5 μL. Mass spectrometry was operated in negative ion mode, and the ionization voltage was 4,500 V, the drying gas flow was 10 L/min, and the gas temperature was 200°C. The nebulizer pressure was set to 40 psig, and the fragment voltage was set to 115 V. Data were collected in centroid mode, and the mass range was set from m/z 40 to 1,000 using an extended dynamic range.

### 2.7 Data and statistical analysis

The data were presented as mean ± standard deviation (SD). SPSS 25 and GraphPad Prism 9.0 were used for *t-*tests and one-way ANOVA for all data analysis. Statistical significance was determined at a *p*-value of < 0.05.

## 3 Results

### 3.1 The monomeric phenolic composition of YAPE

The monomeric phenolic composition of YAPE is presented in Table 1, which includes chlorogenic acid, phloridzin, hyperoside, phloretin, and epicatechin, with respective contents of 38.63, 14.14, 12.53, 12.07, and 10.34%.

**Table 1:** The content of YAPE.

Free phenols	Retention time (min)	Molar mass (%)
Chlorogenic acid	27.15 ± 0.017	38.63
Phloridzin	67.22 ± 0.005	14.14
Hyperoside	65.29 ± 0.005	12.53
Phloretin	78.78 ± 0.008	12.07
Epicatechin	38.32 ± 0.047	10.34
Procyanidine B1	17.55 ± 0.014	4.71
Quercetin	77.12 ± 0.009	3.59
Rutin	66.47 ± 0.005	1.62
Procyanidine B2	25.11 ± 0.271	1.32
Catechin	21.80 ± 0.017	0.61
P-hydroxytoluic acid	20.86 ± 0.014	0.12
Quercitrin	70.09 ± 0.009	0.09
Caffeic acid	29.63 ± 0.014	0.07
Gallic acid	7.62 ± 0.012	0.04

### 3.2 YAPE supplementation improved glycolipid metabolism in diabetic mice

**Figure 1:**
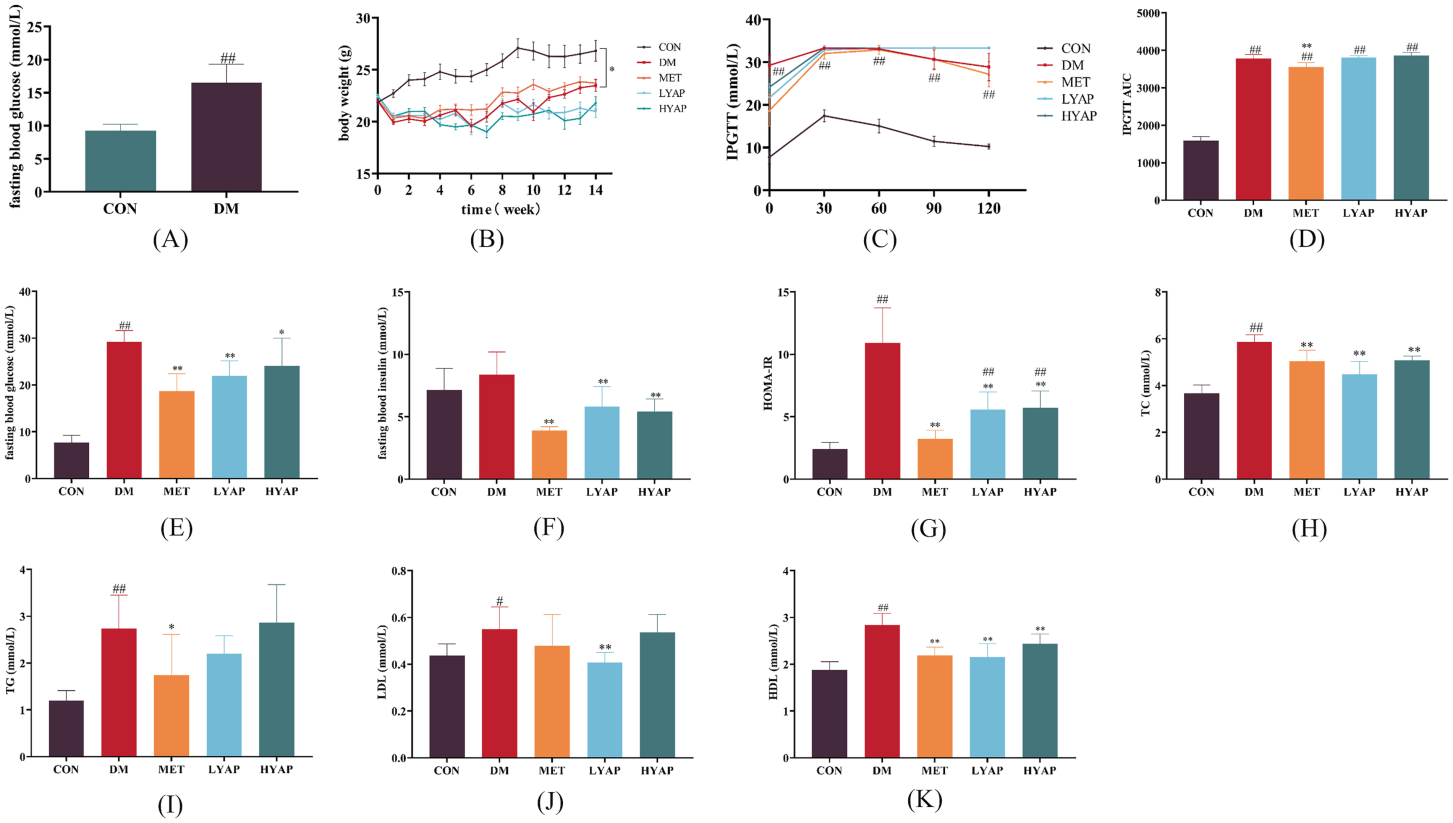
Effects of YAPE on body weight and glycolipid metabolism of diabetic mice. **(A)** Fasting blood glucose in the mice after STZ injection for 5 days; **(B)** body weight; **(C)** intraperitoneal glucose tolerance test (IPGTT); **(D)** AUC of the IPGTT; **(E)** fasting blood glucose; **(F)** fasting blood insulin; **(G)** homeostasis model assessment of insulin resistance (HOMA-IR); **(H)** total cholesterol (TC) in the serum; **(I)** triglyceride (TG) in the serum; **(J)** low-density lipoprotein (LDL); **(K)** high-density lipoprotein. Data are expressed as mean ± SD (*n* ≥ 4). Compared to the DM group, *p < 0.05, **p < 0.01. Compared to the CON group, #*p* < 0.05, ##*p* < 0.01.

Compared to the mice in the CON group, significantly higher levels of FBG were observed in the STZ-induced mice (Figure 1A), indicating the successful establishment of the diabetes model. The mice in the CON group exhibited significantly higher body weight compared to those in the other four groups, whereas supplementation with YAPE did not yield any obvious effect on the body weight of diabetic mice (Figure 1B). The IPGTT results revealed a significant decrease in the area under the IPGTT curve in the CON group compared to the other four groups; however, supplementation with YAPE did not demonstrate any obvious impact on the IPGTT curve (Figures 1C,D). Furthermore, the levels of FBG, fasting insulin, TC, and HDL-C and HOMA-IR were significantly higher in the DM group than in the CON group, but these increases were effectively blocked by the MET, LYAP, and HYAP groups (Figures 1E–H,K). In addition, serum TG and LDL-C levels were significantly higher in the DM group compared to the CON group, but these levels significantly decreased only in the MET and LYAP groups, respectively (Figures 1I,J). Collectively, these data suggest that supplementation with YAPE effectively improved insulin resistance and glycolipid metabolism disorders in diabetic mice.

### 3.3 YAPE supplementation prevented cognitive dysfunction in diabetic mice

**Figure 2:**
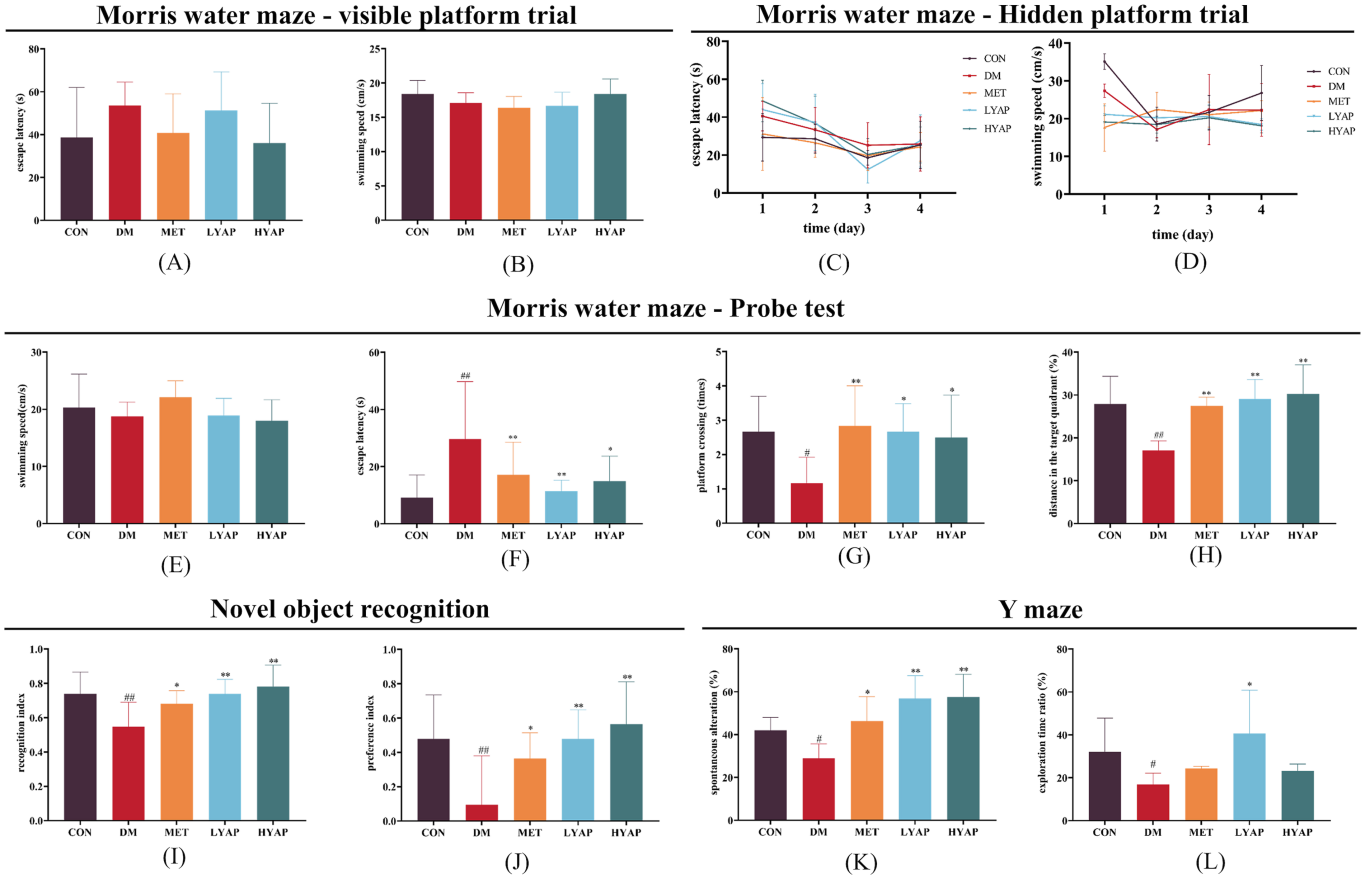
Effects of YAPE on cognitive dysfunction in diabetic mice. **(A)** Escape latency and **(B)** swimming speed of the mice among the different groups in the visible platform trial of the Morris water maze test; **(C)** escape latency and **(D)** swimming speed of the mice among the different groups in the hidden platform trial of the Morris water maze test; **(E)** swimming speed (test period); **(F)** escape latency (test period); **(G)** platform crossing times; and **(H)** distance in the target quadrant (%) of the mice among the different groups in the probe trial of the Morris water maze test. **(I)** recognition index and **(J)** preference index of the mice among the different groups in the NOR test. **(K)** spontaneous alteration (%) and **(L)** exploration time ratio (%) of the mice among the different groups in the Y-maze test. Data are expressed as mean ± SD (*n* ≥ 4). Compared to the DM group, *p < 0.05, **p < 0.01. Compared to the CON group, #*p* < 0.05, ##*p* < 0.01.

After 12 weeks of continuous YAPE supplementation, we evaluated the cognitive function of the mice through a series of behavioral tests. The results of the Morris water maze test showed no significant difference in escape latency and swimming speed during both the visible platform trial and the hidden platform trial (Figures 2A–D). However, during the test period, the mice in the DM group exhibited a significantly higher escape latency compared to the mice in the other four groups (Figure 2F), while their swimming speed remained comparable among all groups (Figure 2E). In addition, there was a notable decrease in both distance in the target quadrant and platform crossings observed in the DM group compared to the other four groups (Figures 2G,H). Furthermore, the findings from the NOR test revealed a significant decrease in both the preference index and recognition index in the mice in the DM group compared to the mice in the other four groups (Figures 2I,J). The results from the Y-maze test indicated a noticeable reduction in the spontaneous alteration and exploration time ratio in the DM group compared to the CON group, whereas it was higher in the LYAP and HYAP groups as compared to the DM group (Figures 2K,L). Collectively, the findings from the behavioral tests suggested that YAPE supplementation had a significant protective effect on learning and memory performance in diabetic mice.

### 3.4 YAPE supplementation inhibited neuronal apoptosis in the cerebral cortex of diabetic mice

**Figure 3:**
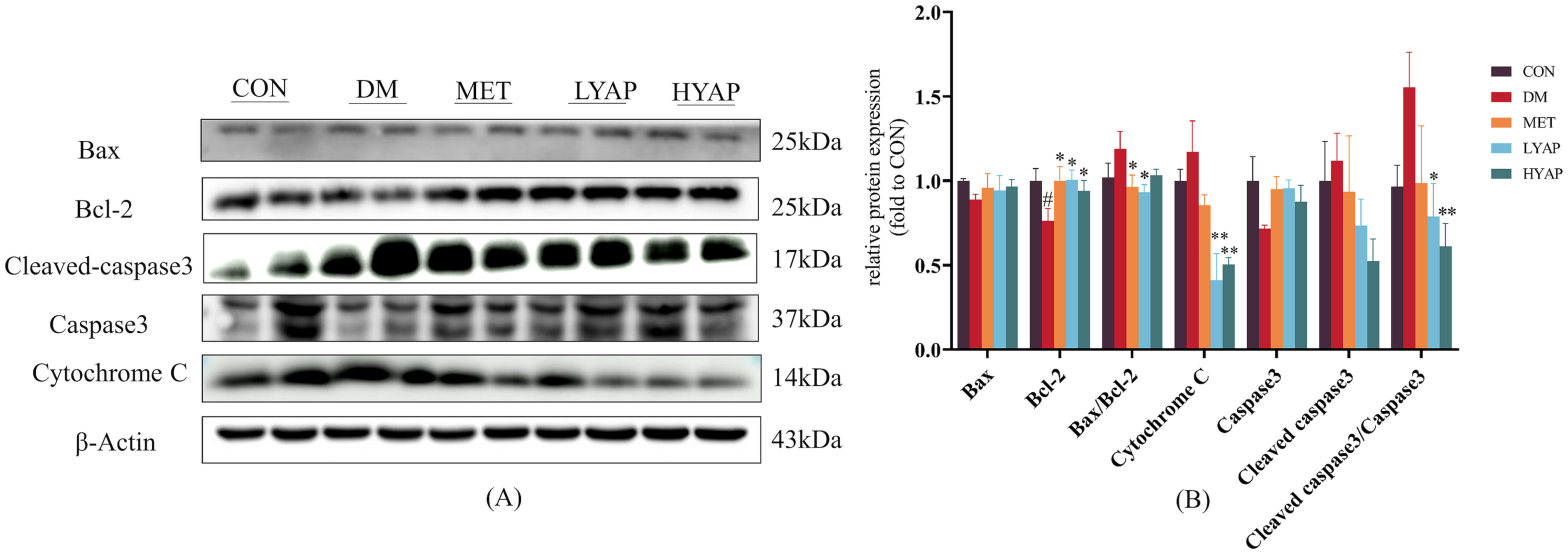
Effects of YAPE on apoptosis-related proteins in the cerebral cortex of diabetic mice. **(A)** Western blot images of apoptosis-related proteins; **(B)** statistical analysis of BAX, Bcl-2, cleaved caspase 3, caspase 3, and cytochrome C. Data are expressed as mean ± SD (*n* ≥ 4). Compared to the DM group, *p < 0.05, **p < 0.01. Compared to the CON group, #*p* < 0.05, ##*p* < 0.01.

Previous studies have demonstrated that neuronal apoptosis in the brain plays a pivotal role in the pathological mechanisms underlying DCD (23). As depicted in Figure 3, there was a significant decrease in the protein expression of Bcl-2 in the DM group compared to the other four groups. Similarly, the ratio of BAX/Bcl-2 in the LYAP and MET groups was lower than that in the DM group. Furthermore, there was a notable reduction in the protein expressions of cytochrome c and the ratio of cleaved caspase 3/caspase 3 in the cerebral cortex of mice belonging to the LYAP and HYAP groups compared to those in the DM group. However, no significant differences were observed in the protein expression levels of BAX, caspase 3, or cleaved caspase 3 among all groups. Consistent with previous findings, these results demonstrate that YAPE supplementation effectively mitigates cognitive dysfunction by inhibiting neuronal apoptosis in the cerebral cortex of diabetic mice.

### 3.5 YAPE supplementation promoted TET2-mediated active DNA demethylation in the cerebral cortex of diabetic mice

**Figure 4:**
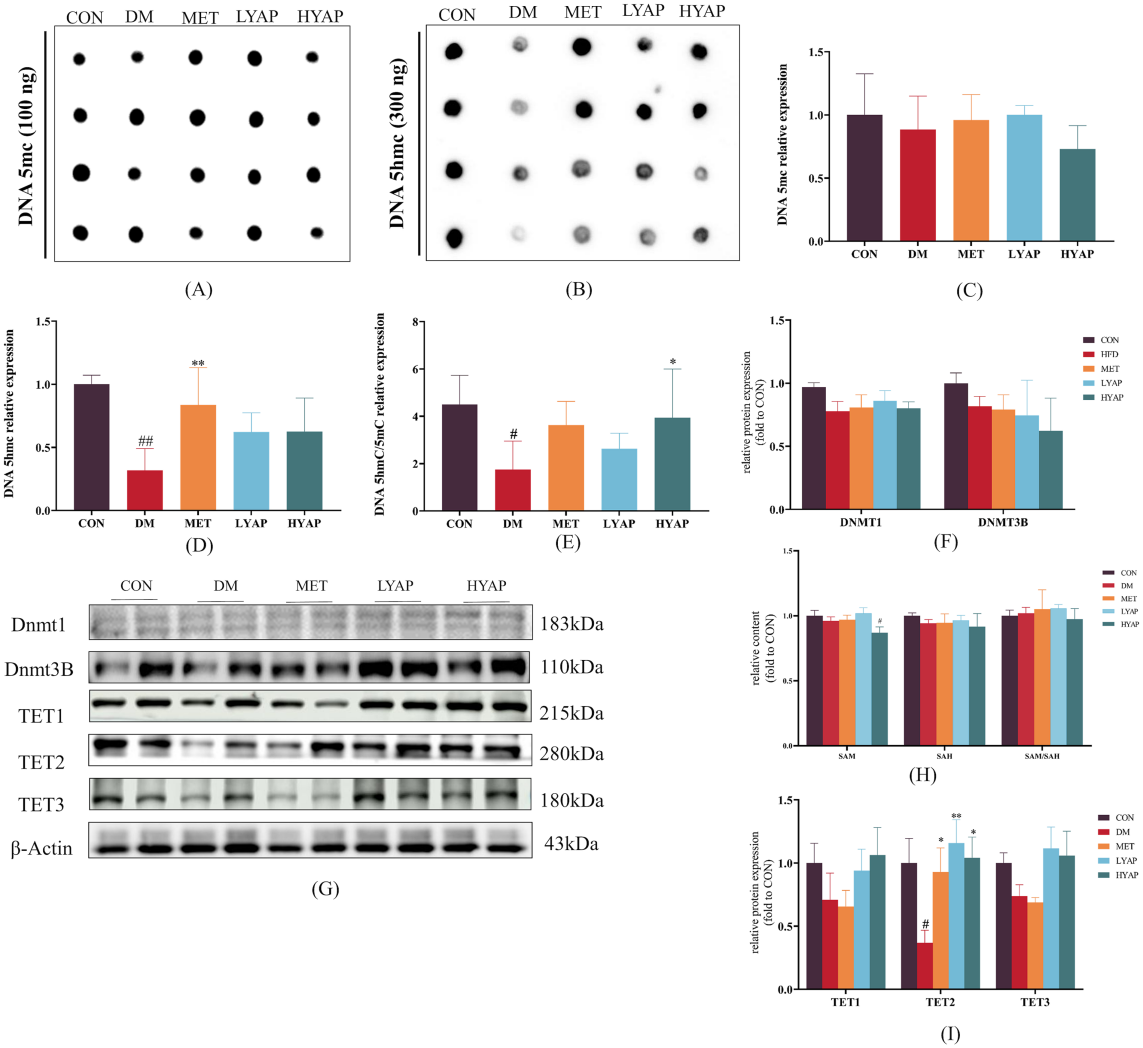
Effects of YAPE on DNA epigenetic metabolism in the cerebral cortex of diabetic mice. **(A)** Dot blot imaging of 5mC; **(B)** dot blot imaging of 5hmC; **(C)** statistical analysis of 5mC; **(D)** statistical analysis of 5hmC; **(E)** the ratio of 5hmC/5mC; **(F)** statistical analysis of DNMT protein levels; and **(G)** Western blot imaging of DNMT1, DNMT3B, TET1/2/3; **(H)** the concentration of SAM and SAH and the ratio of SAM/SAH; **(I)** Western blot images of TET1/2/3 proteins. Data are expressed as mean ± SD (*n* ≥ 3). Compared to the DM group, *p < 0.05, **p < 0.01. Compared to the CON group, #*p* < 0.05, ##*p* < 0.01.

Our previous studies found that decreased DNA demethylation plays a key role in the pathological mechanisms of DCD (5, 11). Therefore, we evaluated the levels of 5mC and 5hmC (the primary products of DNA demethylation) in the cerebral cortex of mice and found no significant differences in the levels of 5mC among all groups (Figures 4A,C). Meanwhile, a significant decrease was observed in the levels of 5hmC in the DM group compared to the CON and MET groups (Figures 4B,D), along with a significantly lower ratio of 5hmC/5mC in the DM group compared to the CON and HYAP groups (Figure 4E). To elucidate the underlying mechanisms responsible for the alterations in 5hmC, we examined the dynamics of DNA epigenetic modification metabolism regulators, including DNMT and TET enzymes, and the concentrations of SAM and SAH in the cerebral cortex of diabetic mice. The Western blot analysis revealed no significant differences in DNMT1 and DNMT3B protein levels across all experimental groups (Figures 4F,G). Given that SAM serves as a methyl donor and plays a crucial role in DNA methylation, we subsequently assessed the levels of SAM and its demethylation product, SAH. Similarly, no significant differences were observed in the SAH concentration or SAM/SAH ratio across all groups; however, the HYAP group exhibited a significantly lower SAM concentration compared to the CON group (Figure 4H). The TET family of proteins catalyzes the conversion of DNA 5mC to DNA 5hmC (24). We assessed the expression of TET family proteins and found that only the TET2 protein level was significantly reduced in the DM group compared to the other experimental groups (Figures 4I,J).

### 3.6 YAPE supplementation improved TET2 function in the cerebral cortex of diabetic mice

**Figure 5:**
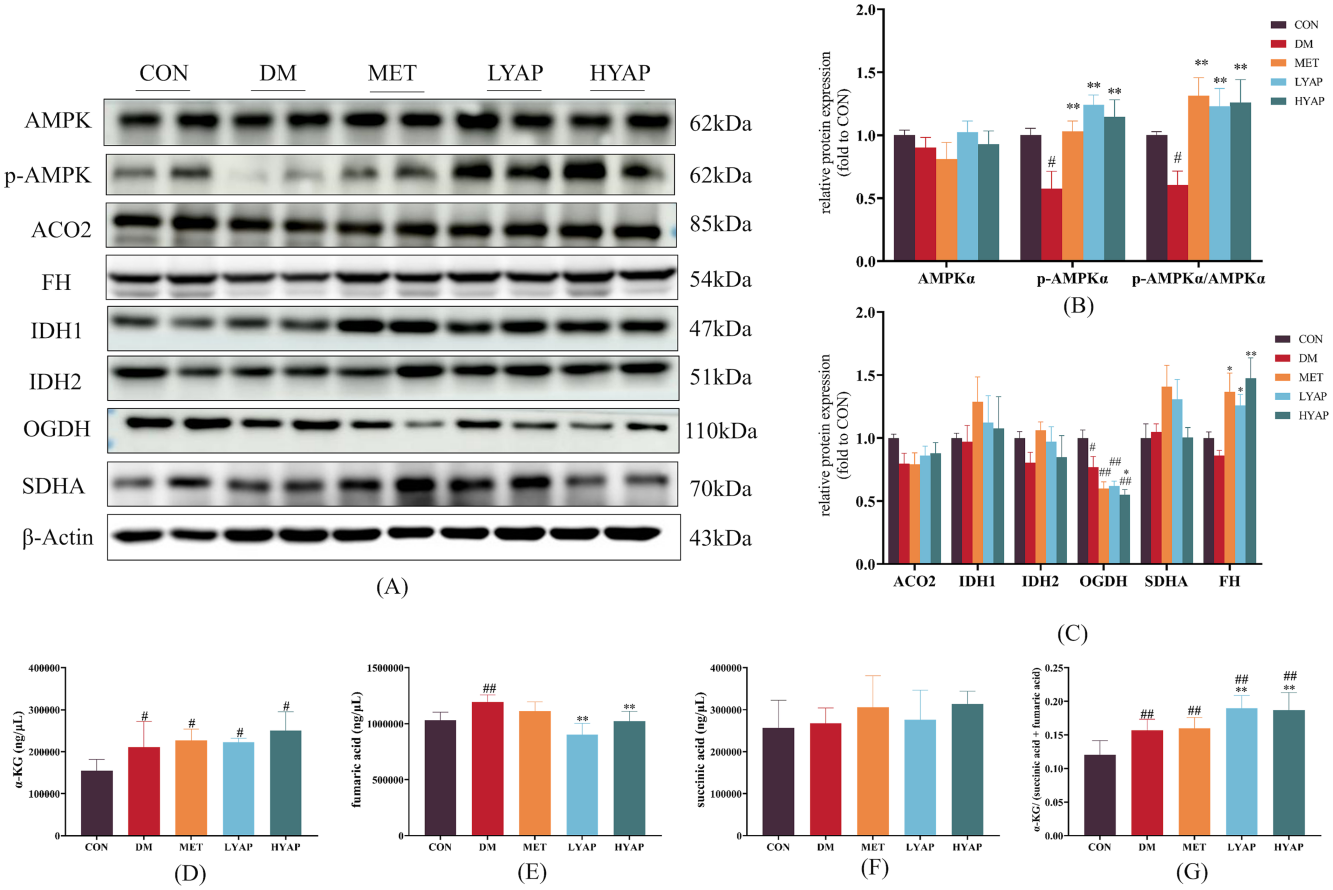
Effects of YAPE on the regulators of TET2 function in the cerebral cortex of diabetic mice. **(A)** Western blot imaging of AMPK and TCA metabolites; **(B)** statistical analysis of AMPK protein levels; **(C)** statistical analysis of TCA metabolites; **(D)** the content of α-KG in the cerebral cortex of mice; **(E)** the content of fumaric acid in the cerebral cortex of mice; **(F)** the content of succinic acid in the cerebral cortex of mice; and **(G)** the ratio of α-KG/(succinic acid + fumaric acid) in the cerebral cortex of mice. Data are expressed as mean ± SD (*n* ≥ 4). Compared to the DM group, *p < 0.05, **p < 0.01. Compared to the CON group, #*p* < 0.05, ##*p* < 0.01.

The protein stability of TET2 is regulated by AMPK (9). Therefore, we examined the protein levels of p-AMPK and AMPK and found a significant decrease in both p-AMPK levels and the p-AMPKα/AMPKα ratio in the DM group compared to the CON group. Importantly, YAPE supplementation led to an increase in both p-AMPK levels and the p-AMPK/AMPK ratio (Figures 5A,B). In addition, we investigated the protein levels of TCA cycle-related enzymes in the cerebral cortex of mice. The results revealed a significant increase in the protein levels of α-ketoglutarate dehydrogenase (OGDH) in the CON group compared to the other groups (Figures 5A,C). Furthermore, the protein level of fumarate dehydrogenase (FH) was significantly elevated in the MET, LYAP, and HYAP groups compared to the DM group, while no significant differences were observed in the levels of other proteins, including aconitase 2 (ACO2), isocitrate dehydrogenase (IDH), and succinate dehydrogenase (SDH), among all groups (Figures 5A,C). In addition, we analyzed the concentration of TCA cycle intermediates in the cerebral cortex of mice. The results revealed a significant decrease in the concentration of α-KG in the CON group compared to the other groups (Figure 5D). Conversely, the fumaric acid level was significantly higher in the DM group than in the CON, LYAP, and HYAP groups (Figure 5E). No significant differences were observed in the levels of succinic acid among all groups (Figure 5F). In addition, we found that the α-KG/(succinic acid + fumaric acid) ratio was significantly lower in the DM group compared to the LYAP and HYAP groups (Figure 5G). The findings indicate that YAPE enhances protein stability and enzyme activity to promote TET2 function.

## 4 Discussion

With the aging population and the increasing prevalence of diabetes mellitus among older adults, DCD is expected to emerge as a significant neurological disorder that poses a substantial threat to brain health (25). Population studies have shown that the consumption of apples, apple-rich drinks, or apple extract has beneficial effects on markers related to diabetes or its complications (13, 26, 27). Furthermore, animal studies have revealed that the administration of apple polyphenols not only notably alleviates neuroinflammation in the brains of mice but also enhances impaired cognitive function (19, 28). Similar to the aforementioned findings (29, 30), our study demonstrated that YAPE supplementation effectively ameliorated glycolytic metabolic disorders by improving insulin sensitivity and reducing lipid accumulation in diabetic mice (Figure 1). Notably, we also found a dose-dependent protective effect of YAPE supplementation on cognitive function decline in the diabetic mice (Figure 2). Therefore, it is of great significance to elucidate the precise molecular mechanism underlying the preventive effects of YAPE on DCD.

Excessive neuronal apoptosis is a pivotal factor contributing to the onset and progression of DCD (31). The combined use of STZ injection and a high-fat diet has been reported to cause diabetic cognitive dysfunction and mitochondrial damage, often resulting in DCD (32–34). Previous studies have demonstrated that an apple polyphenol mixture or monomeric phenols derived from apples mitigates excessive neuronal apoptosis in the brains of mice. The protein levels of cytochrome c and a reduced ratio of cleaved-caspase 3/caspase 3 in the cerebral cortex of mice in LYAP and HYAP groups were lower compared to the DM group (Figure 3). These results indicate that YAPE exhibits a promising protective effect on cognitive function by suppressing neuronal apoptosis in the brains of diabetic mice.

Numerous studies have established that dysregulation of DNA epigenetic homeostasis is a crucial contributor to neuronal apoptosis and the development of DCD (5, 10, 11, 35). In addition, 5hmC serves as the primary intermediate in the TET-mediated active DNA demethylation process and exerts significant regulatory effects on neurodevelopment and the maintenance of brain function (24, 36, 37). The present study observed a significant decrease in the ratio of 5hmC/5mC in the cerebral cortex of mice in the DM group compared to those in the CON group, which was effectively ameliorated by YAPE supplementation (Figures 4A–E). Furthermore, our previous studies have indicated that the upregulation of 5hmC levels in the cerebral cortex of diabetic mice serves as a potential inhibitory mechanism for neuronal apoptosis (5, 10, 12).

To further investigate the molecular mechanisms underlying YAPE-mediated regulation of DNA epigenetic homeostasis, we assessed the changes in pertinent regulators involved in DNA methylation and demethylation. As it is well known, DNMTs catalyze DNA methylation using SAM as methyl group donors and SAH as methyl group receptors (38). The ratio of SAM to SAH serves as an indicator of DNA methylation levels (39). Our results revealed no significant differences in the protein levels of DNMT1 and DNMT3B, nor in the SAM/SAH ratio, among the groups, which also partly confirmed that there were no significant differences in DNA 5mC levels in the cerebral cortex of mice (Figures 4F–H). The process of active DNA demethylation is mainly dependent on TET function, and dysfunction of TET2 is a significant contributor to cognitive dysfunction (40). The results of our study demonstrated significant upregulation of TET2 protein levels in the cerebral cortex of diabetic mice following YAPE supplementation (Figure 4G). Previous studies have demonstrated that the stability of TET2 protein is regulated through AMPK-mediated phosphorylation at a residue (9). In our current study, we found that the ratio of p-AMPK/AMPK is higher in the other four groups compared to the DM group (Figures 5A,B). In addition, TET enzyme activity is positively regulated by α-KG, while succinate and fumarate competitively inhibit α-KG, thereby suppressing TET enzymatic activity (41–43). In the current study, we observed that YAPE enhanced the homeostasis of the TCA cycle in the cerebral cortex of diabetic mice (Figure 5). Previous studies have demonstrated that dysregulated metabolism of DNA epigenetic modifications leads to the regulation of neuronal apoptosis-related gene expression, including BAX and Bcl-2, in the pathogenesis of neurodegenerative disease (44, 45). In conjunction with the aforementioned evidence, our findings suggest that YAPE enhances TET2-mediated active DNA demethylation by improving the homeostasis of the TCA cycle and AMPK-mediated phosphorylation in the cerebral cortex of diabetic mice (Figure 5).

It is widely known that metformin, a first-line antidiabetic agent extensively utilized in clinical practice, exhibits a variety of preventive effects against diabetic complications (46, 47). In this study, we used metformin (150 mg/kg) as a positive control to investigate the biological activity of YAPE. It is worth noting that YAPE exhibited a more pronounced enhancement in cognitive function compared to metformin in diabetic mice (Figure 2). As outlined in the introduction, to ensure both the yield and quality of apples, approximately 2 million tons of young apples are culled annually in China. This practice provides abundant and cost-effective raw materials for YAPE production. All of these factors will collectively establish a robust and comprehensive foundation for the application of YAPE in the prevention and management of DCD. However, there are several limitations in our study that should be noted. First, although we found that YAPE prevents DCD by promoting TET2-mediated active DNA demethylation in the brains of diabetic mice, the precise molecular mechanisms remain to be determined. Second, the neuro apoptosis-related genes regulated by TET2-mediated active DNA demethylation remain unclear. Future studies should use genome-wide sequencing of DNA methylation or hydroxymethylation to identify the specific genes regulated by YAPE. Third, it is important to note that YAPE is a mixed polyphenolic compound, and the composition of monomeric phenols may vary across different batches. This variation could potentially result in inconsistencies in its biological effects. Consequently, the standardization of quality control for YAPE is one of the critical issues that must be urgently addressed to facilitate its clinical application.

In summary, the process of TET2-mediated active DNA demethylation is impaired in the brains of diabetic mice, leading to abnormal expression of neuronal apoptosis-related genes and subsequently promoting the onset of DCD. Conversely, YAPE exerts its protective effects by enhancing TET2 function through the improvement of the homeostasis of the TCA cycle and phosphorylated AMPK, thereby preventing the occurrence of DCD. The present study elucidated the molecular mechanism underlying the preventive effect of YAPE on DCD from an epigenetic perspective, providing a novel nutritional intervention strategy for DCD prevention.

## Data Availability

The datasets presented in this study can be found in online repositories. The names of the repository/repositories and accession number(s) can be found in the article/supplementary material.
